# Accuracy of Panoramic Radiograph for Diagnosing Periodontitis Comparing to Clinical Examination

**DOI:** 10.3390/jcm9072313

**Published:** 2020-07-21

**Authors:** Vanessa Machado, Luís Proença, Mariana Morgado, José João Mendes, João Botelho

**Affiliations:** 1 Periodontology Department, Clinical Research Unit (CRU), Centro de Investigação Interdisciplinar Egas Moniz (CiiEM), Instituto Universitário Egas Moniz (IUEM), 2829-511 Almada, Portugal; jbotelho@egasmoniz.edu.pt; 2 Clinical Research Unit (CRU), CiiEM, IUEM, 2829-511 Almada, Portugal; mmorgado@egasmoniz.edu.pt (M.M.); jmendes@egasmoniz.edu.pt (J.J.M.); 3 Quantitative Methods for Health Research (MQIS), CiiEM, IUEM, 2829-511 Almada, Portugal; lproenca@egasmoniz.edu.pt

**Keywords:** periodontal disease, periodontitis, periodontal bone loss, periodontitis cases screening, panoramic dental X-ray

## Abstract

In this study, we explore the diagnostic accuracy of a Radiographic-based Periodontal Bone Loss (R-PBL) method as a screening tool for periodontitis, in the form of radiographic bone loss, under the 2018 case definition in comparison to the 2012 case definition. The analysis was based on 456 patients (253 females and 203 males), screened for periodontal status in the Study of Periodontal Health in Almada-Seixal (SoPHiAS) project and subjected to a panoramic dental X-ray. Patients were diagnosed for the presence of periodontitis following the 2018 and 2012 case definition. R-PBL classification was defined by alveolar bone loss and diagnosed as no periodontitis (≥80% remaining alveolar bone), mild to moderate periodontitis (66% to 79%), or severe periodontitis (<66%). We appraise the X-ray quality to look for the influence on the performance of R-PBL. Sensitivity, specificity, accuracy, and precision, through several indicators, were determined. Performance measurement was assessed through binary and multiclass Receiver operating characteristic/are under the curve (ROC/AUC) analyses. Our results show that the tested R-PBL method under the 2018 case definition is a reliable tool in periodontitis cases screening. This method does not replace clinical periodontal evaluation, but rather, it screens patients towards a definitive periodontitis diagnosis. These results will contribute to support the development of automated prediction systems towards periodontitis surveillance.

## 1. Introduction

Over the past few years, several surveillance programs have implemented different methods to assess periodontal status [1,2]. Periodontal diagnosis using Full-mouth Recording Protocols (FRP) is considered the “gold standard” to determine individual periodontal status [3,4,5,6,7]. Nevertheless, full-mouth examination requires the assessment of several parameters and the recording of a great deal of information [8]. Besides, FRP is very demanding in terms of time and effort both for patients and for examiners, which could result in measurement errors and large dropout rates [3,9].

The diagnosis of periodontitis is based mainly on clinical examination [8]. Still, radiographic assessment is a critical component that confirms the presence of interproximal clinical findings of periodontal bone levels to estimate the prognosis of periodontally involved teeth, the treatment plan and the evaluation of the recurrence or progression of periodontitis [10,11]. In this sense, radiographic bone loss evaluation becomes particularly important to the classification of periodontitis based on stages defined by severity, and grades that reflect this disease progression [10].

Panoramic radiograph is a routine dental care imaging method that provides a valuable screening opportunity [12]. This two-dimensional radiography provides important and additional information which could potentially guide during periodontal staging and grading. In particular, a panoramic radiograph is useful in the measurement of Periodontal Bone Loss (PBL) [13], and intraoral and panoramic radiographic PBL measurements have been demonstrated to be clinically coincident [14]. Additionally, PBL was successfully validated as a reliable tool for large-based surveillance studies in Sweden, in particular the periodontitis and its relation to coronary artery disease (PAROKRANK), a multicenter case-control study, recruiting patients at 17 Swedish hospitals [15,16,17]. Furthermore, radiographic-based PBL measurements in machine-learning-based technologies had very good performance and equal discrimination as dentists [13]. However, all these methods shall follow up-to-date case definitions and the performance of radiographic-based PBL methods using the 2018 case definition remains to be elucidated.

The present study aimed to explore the diagnostic accuracy of a Radiographic-based PBL (R-PBL) method as a screening tool for periodontitis, in the form of radiographic bone loss, according to the 2018 case definition in comparison to the 2012 case definition.

## 2. Materials and Methods

### 2.1. Study Design and Eligibility Criteria

The Study of Periodontal Health in Almada-Seixal (SoPHiAS) is a population-based representative study that recruited 1064 individuals from December 2018 to April 2019 [18]. All participants gave their previous written informed consent. Patients received full-mouth periodontal diagnosis at their respective Health Center, as previously reported [18]. All patients were referred to Egas Moniz Dental Clinic (EMDC) for a free full dental check-up with a panoramic radiograph and dental cleaning. Furthermore, periodontitis patients had the opportunity to undergo periodontal treatment without costs at the EMDC. In the EMDC, before the examination, patients signed an informed consent form. To be eligible for participation in this study, the SoPHiAS participant had to consent to participate and undergo a panoramic radiograph in the EMDC as a result of the free available follow-up care.

This study was approved by two state-recognized ethics committees: the Research Ethics Committee of the Regional Health Administration of Lisbon and Tagus Valley, IP (Registration numbers: Process: 3525/CES/2018 and 8696/CES/2018) and the Ethics Committee of Egas Moniz (Registration numbers: Process: 595). This study followed the Standards for Reporting Diagnostic Accuracy (STARD) recommendations [19] (Appendix A).

### 2.2. Radiographic-Based Periodontal Bone Loss (PBL) Method

**Figure 1:**
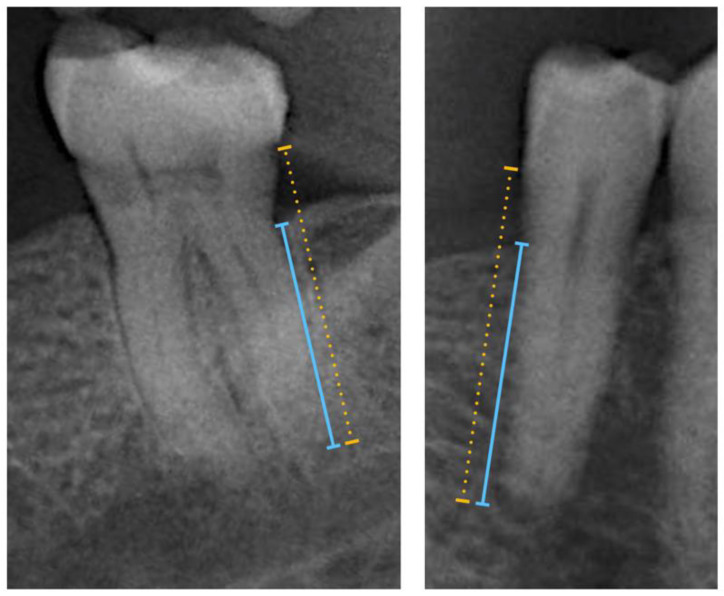
Radiographic-based Periodontal Bone Loss (R-PBL) was measured from the marginal alveolar bone to the tooth apex (blue line) and from the cementoenamel junction to the tooth apex (yellow dotted line), as in Rydén et al. [16] The examples show R-PBL measurement in a molar and premolar.

Radiographic examination followed the standardized protocol by Rydén et al. [16] Third molars were excluded, accounting for a possible total of 28 teeth. Dental implants were not examined. Dentures, complete, partial, and a complete implant bridge, in either jaws, were classified as removable dentures [16]. Panoramic radiographs were taken using the digital Orthophos XG 5 DS/Ceph (Sirona Dental System, New York, NY, USA) at the Radiology Department at the EMDC. Three calibrated and blinded examiners (VM, JB and MM) used ImageJ (Image Tool 3.0 software program, Department of Dental Diagnostics Science, University of Texas Health Science Center, San Antonio, TX, USA) for the periodontal diagnosis through radiographic-based PBL measurements. The PBL was assessed by measuring the total root length (distance from the tooth’s apex to the cementoenamel junction) and the total bone height (distance from the tooth’s apex to the marginal bone crest), in each tooth (Figure 1). Measurements were performed with a high-resolution computer monitor in a darkened room. For these measurements, the arithmetic mean was then calculated and used as a measure of proportion (%). Based on the PBL, in percentage, patients were then divided into different groups: Healthy Periodontium (if PBL ≥ 80%), Mild-to-Moderate Periodontitis (if PBL was ranged between 79 and 66%), and Severe Periodontitis (if PBL < 66%) [16].

### 2.3. Panoramic Radiographs Quality Assessment

Qualitative assessment of panoramic radiographic quality was assessed by one researcher (MM), following the criteria proposed by Sabarudin and Tiau [20]. The zones of assessment were adapted for two areas, the upper and lower arches. For both jaws, the ordinal grading scale addressed (1) anatomy coverage, (2) density and contrast, and (3) anatomical structures [20]. In a situation of teeth absence or presence of implants, only the term Non-Applicable (NA) was applied. The panoramic radiographs were rated as high quality if all scores were graded 3 or 4. If, at least, one score was graded 1 or 2, then a low quality was assigned.

### 2.4. Measurement Reliability and Reproducibility

Panoramic radiographs were obtained by a certified radiologist using the Orthophos XG 5 DS/Ceph (Sirona Dental System, New York, USA) according to the manufacturer’s instructions, and stored at an informatic software system database Sidexis (Sirona, Bensheim, Germany). Two trained and calibrated examiners (VM and JB) performed the periodontal examination and data collection as previously detailed [18]. Intra-class correlation coefficient (ICC) were 0.98 and 0.99 for Clinical Attachment Loss (CAL) and Probing Depth (PD), respectively. Intra-examiner ICC ranged from 0.97 to 0.99, for both PD and CAL, respectively.

For radiography assessment calibration purposes, three examiners (VM (dentist 1), JB (dentist 2) and MM (dentist 3)) assessed 30 randomly selected panoramic radiographs, in a total of 90 separate observations. The three examiners were in complete agreement in 28 of 30 observations (93.3%). The correlation between dentist 1 and 2 was 0.93, the correlation between 1 and 3 was 0.95, and the correlation between 2 and 3 was 0.97 (κ value, 0.90).

### 2.5. Data Management, Test Methods and Analysis

The results of the PBL (%) measurements after the categorization into Healthy Periodontium, Mild-to-Moderate and Severe Periodontitis were compared to the same patient’s periodontal diagnosis following the Classification of Periodontal Diseases and Conditions, by the American Academy of Periodontology (AAP) [21] and the Classification of Periodontal and Peri-implant Diseases and Conditions, by the AAP and the European Federation of Periodontology (EFP) [8], in order to identify the accuracy of the panoramic radiograph periodontitis diagnosis. For each periodontal case definition, specific Microsoft Office Excel datasets were derived in order to formulate appropriate algorithms.

In the performance analysis, full-mouth diagnosis was used as the standard reference for each case definition because it represents entirely the periodontal status. To test the index performance, we started by computing the final diagnosis into two variables according to the presence of disease (coded: 0—no, 1—yes) and the staging (coded: 0—non periodontitis, 1—mild, 2—moderate, 3—severe). Then, contingency tables were used to calculate true positive (TP), true negative (TN), false positive (FP) and false negative (FN) values. From this, sensitivity, specificity, accuracy and precision, through several indicators, were determined (Table 1) [22]. Data was handled with Microsoft Office (MO) Excel. Moreover, Diagnostic Odds Ratio (DOR) and the respective Standard Error (SE) and 95% Confidence Interval (95% CI) were estimated. Performance measurement was assessed through binary and multiclass Area Under the Curve (AUC), through Receiver Operating Characteristics (ROC) analysis. For AUC/ROC analysis, we used the R package “plotROC” [23] (by means of “roc” and “multiclass.roc” functions. Data was analyzed as originally recorded, without missing data handling.

## 3. Results

### 3.1. Participant Characteristics

**Figure 2:**
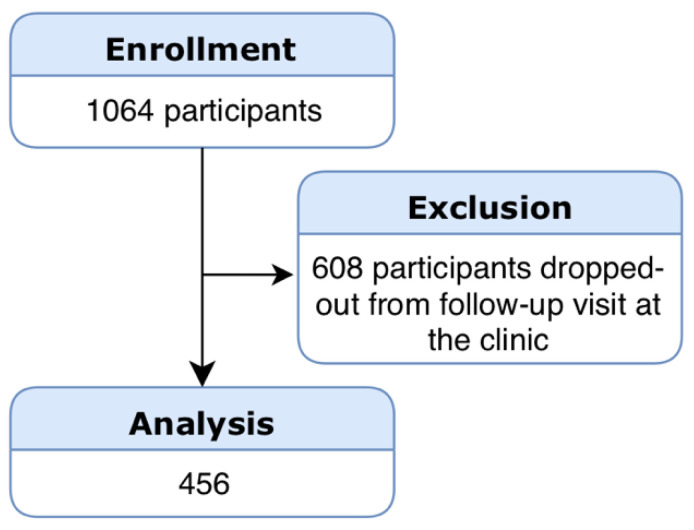
Flowchart of participants.

From an initial sample of 1064 individuals, 456 consecutive patients (aged 18 to 89 years old) according to eligibility criteria were enrolled in the study (Figure 2). One hundred and eighty-five patients had no disease, 160 had mild-to-moderate periodontitis and 111 patients were diagnosed with severe periodontitis according to EFP/AAP 2018 case definition. The demographic and clinical characteristics of participants with the periodontitis staging distribution are depicted in Table 2.

### 3.2. Accuracy Performance of R-PBL for the Presence of Periodontitis

Concerning the presence of periodontitis, R-PBL through the 2018 case definition outperformed the 2012 case definition (Table 3). In terms of sensitivity and sensibility, R-PBL showed better performance under the 2018 case definition, and higher accuracy, Youden’s index, F1 and MCC scores. The AUC and Precision values were more favorable for the 2012 case definition than for the 2018 classification. In both classifications, R-PBL was shown to be a sensitive method towards the radiographic quality score.

### 3.3. Accuracy Performance of R-PBL for the Staging of Periodontitis

Multiclass ROC analyses, using AUC values, showed that the 2018 case definition has slightly improved the performance of R-PBL in the staging of periodontitis without considering the radiographic score (Table 4). Remarkably, R-PBL was shown to be very sensitive to the radiographic quality score when employed with the 2018 classification, while for the 2012 case definition, this difference was less pronounced. Furthermore, R-PBL methodology on high-quality panoramic radiographs with the 2018 classification was the only strategy that obtained good accuracy as a diagnostic test, while the remaining had fair accuracy.

## 4. Discussion

We have theorized that R-PBL might be a competent screening tool for periodontitis for epidemiologic surveillance purposes. To examine this hypothesis, we compiled a representative sample from the SoPHiAS study that has been fully diagnosed periodontally and has undergone a panoramic radiograph at the EMDC. Then, we performed the tested R-PBL in the panoramic radiographs and compared the results with the 2018 and 2012 case definitions. Our results validate this R-PBL method as a reliable and valid instrument in the surveillance of periodontitis cases.

Our findings have potential worldwide implications. (1) From a population-based surveillance standpoint, the R-PBL method following the 2018 periodontal case definition presents a prospective advantage towards oral public health watch. (2) Periodontitis is a global public health challenge due to its worrisomely global prevalence and has serious health, economic, and quality-of-life burdens [24]. (3) Periodontitis surveillance programs are scarce, mainly because they are very demanding in terms of time, workforce, and money; using this strategy may overcome these difficulties. (4) Therefore, this method might contribute to enhance the number of population-based surveillance studies on periodontitis in a large-scale scenario.

Undisputedly, panoramic radiographs provide diagnostic value to periodontology on radiographic bone levels, plaque retention factors, caries lesions, furcation lesions, subgingival calculus, and other conditions [25,26,27]. With the 2018 update [8,10,28], despite radiographic imaging being less sensitive to assess periodontal tissues collapse, it is now considered sufficient to establish staging of the disease after clinical confirmation of a periodontitis case. This is a very relevant fact at the clinical level, because on the one hand it allows periodontal screening to assess the possibility of the patient presenting patterns of periodontal destruction and thus forwarding it to the periodontologist. On the other hand, it allows to adequately monitor a periodontal patient after periodontal treatment and, thus, to re-signal this patient in a situation where there is a lack of control.

Interestingly, recent studies have strengthened the clinical and epidemiologic dimensions of the new 2018 case definition. Firstly, this new case definition proved to be a consistent instrument to portray patients’ clinical characteristics, disease progression, and eventual tooth loss [29]. Secondly, the 2018 case definition outperformed the previous 2012 case definition in terms of diagnosis and staging of periodontitis on full-mouth partial recording protocols, which has a substantial epidemiologic potential [7]. From our results, the 2018 diagnosis system additionally increases the ability to diagnose patients towards periodontitis at screening surveillances, in order to refer patients to specialized periodontology consultations.

Our results corroborate the potential implications for public health prevention programs, since a recent study successfully applied deep convolutional neural networks (CNNs) to detect PBL on panoramic radiographs [13]. The accuracy displayed in our study supports the integration of the up-to-date classification system in the development of automated prediction systems through algorithms in the future. Therefore, the reality of detecting potential periodontitis cases in a surveillance setting using fully automated and informatic resources based in panoramic radiographs may soon become a reality, with an unprecedented potential impact. Another aspect that readers must bear in mind is the fact that this technology may also be individually available in the format of mobile applications if patients keep their panoramic radiographies. In this sense, patients can themselves obtain information if they are at risk of being a periodontitis case and can take appropriate action.

Surveillance is needed to detect potential public health emergencies, as periodontitis is indeed one of them [24]. Collectively, surveillance is warranted to serve as an early warning system, to identify public health emergencies, to strive public health policy and strategies, to report on the impact of an intervention or progress on a particular problem and to learn the epidemiology of a condition under particular characteristics [30]. Public health surveillance relies on an ongoing, systematic collection, analysis, and interpretation of health-related data essential to the planning, implementation, and evaluation of public health practice [30]. Therefore, the validation of R-PBL for periodontitis using up-to-date case definitions is key. Such a validation may be useful to national health systems to introduce new detection systems on periodontal health combined with groundbreaking technologies such as CNN or other artificial intelligence machine-learning tools.

Our study benefits from a strong methodology following international guidelines on diagnostic accuracy reports, a full-mouth periodontal diagnosis and its basis in a sample derived from a representative study. Despite the countless potentialities, there are a number of shortcomings worth mentioning. One of the limitations of the R-PBL method through panoramic radiography is its inability to accurately image the osseous structure three-dimensionally; consequently we might continuously expect a certain level of inaccuracy. A possible alternative could be the integration of cone-beam computerized tomography images in this type of dental imagery diagnostics armamentarium, since periapical and bite-wing radiographs are accepted as the current standard X-ray in periodontal care. Moreover, this method does not diagnose active periodontitis and does not inform about periodontal soft tissues. However, this inherent limitation means that R-PBL can never be seen as an instrument of final diagnosis, but only as an aid in the diagnosis of potential cases of periodontitis in the epidemiological perspective, and thus serves only for its screening. Finally, it is important to note that factors such as patient age, tooth type, or angulation of teeth can all influence alveolar bone height, thus we must be alert for these confounding factors [11].

## 5. Conclusions

From a population-based surveillance standpoint, the tested R-PBL method under the 2018 case definition is a reliable tool in periodontitis case screening. This method is sensitive to the radiographic quality score and does not replace clinical periodontal evaluation, which is indispensable to definitive diagnosis of periodontitis. These results may contribute to supporting the development of automated prediction systems towards periodontitis surveillance.

## Figures and Tables

**Table 1:** Diagnostic performance indicators used in the comparative analysis.

Sensitivity (True Positive Rate)	TP/(TP + FN)	Proportion Positive Test Results Among Diseased
Specificity (True negative rate)	TN/(TN + FP)	Proportion negative test results among the “healthy”
Accuracy	(TP + TN)/(TP + TN + FP + FN)	Proportion of correctly identified subjects
Precision—Positive Predictive Values (PPV)	TP/(TP + FP)	-
Youden’s index	Sensitivity + Specificity − 1	Measures the performance of a dichotomous diagnostic test
DOR	(TP/FN)/(FP/TN)	Ratio of the odds of positivity in disease relative to the odds of positivity in the non-diseased
DOR (95% CI)	95% CI = log DOR ± 1.96SE(log DOR), where $\mathrm{SE}\left(\mathrm{logDOR}\right)=\sqrt{\frac{1}{\mathrm{TP}}+\frac{1}{\mathrm{TN}}+\frac{1}{\mathrm{FP}}+\frac{1}{\mathrm{FN}}}$	-
F1 Score	2TP/(2TP + FP + FN)	Harmonic mean of precision and sensitivity
Matthews Correlation Coefficient (MCC)	(TP × TN − FP × FN)/ $\sqrt{(\left(\mathrm{TP}+\mathrm{FP}\right)\times \left(\mathrm{TP}+\mathrm{FN}\right)\times \left(\mathrm{TN}+\mathrm{FP}\right)\times \left(\mathrm{TN}+\mathrm{FN}\right)}$	Measure of quality of binary classifications

95% CI: 95% Confidence Interval; DOR: Diagnostic Odds Ratio; FN: False Negative, FP: False Positive; SE: Standard Error; TN: True Negative; TP: True Positive. Adapted from [22].

**Table 2:** Demographic and clinical characteristics of participants (*n* = 456), with periodontitis severity distribution according to the EFP/AAP 2018 and CDC/AAP 2012 case definitions.

Variable	Result
Age, mean (SD)	59.9 (15.7)
Gender, *n* (%)	
Female	253 (55.5)
Male	203 (44.5)
Periodontal status (EFP/AAP 2018), *n* (%)	
Healthy	185 (40.6)
Periodontitis	271 (59.4)
Mild–Moderate (Stage I/II)	160 (35.1)
Severe–Advanced (Stage III/IV)	111 (24.3)
Periodontal status (CDC/AAP 2012), *n* (%)	
Healthy	110 (24.1)
Periodontitis	346 (75.9)
Mild–Moderate	218 (47.8)
Severe	128 (28.1)
Missing teeth	9.2 (6.7)
BoP (%)	13.9 (20.0)
Mean PD	1.95 (0.81)
Mean CAL	2.71 (1.47)
Mean Recession	0.77 (0.98)
Panoramic radiograph quality	
Low quality	281 (61.6)
High quality	175 (38.4)

SD: standard deviation; EFP/AAP: European Federation of Periodontology/ American Academy of Periodontology; CDC/AAP: Centers for Disease Control and Prevention/AAP; CAL: Attachment Loss; PD: Probing Depth; BoP: Bleeding on Probing.

**Table 3:** R-PBL performance analysis according to the EFP/AAP 2018 and CDC/AAP 2012 case definitions (*n* = 456). Values are expressed as mean and 95% Confidence Interval (95% CI).

	EFP/AAP 2018			CDC/AAP 2012		
	Overall (*n* = 456)	High-Quality Radiography (*n* = 175)	Low-Quality Radiography (*n* = 281)	Overall (*n* = 456)	High-Quality Radiography (*n* = 175)	Low-Quality Radiography (*n* = 281)
TP	270	103	167	280	100	180
FN	1	0	1	42	12	30
FP	76	25	51	66	28	38
TN	109	47	62	68	35	33
Sensitivity	99.6 (99.1–100)	100.0 (100–100)	99.4 (98.7–100)	87.0 (83.9–90.0)	89.3 (86.4–92.1)	85.7 (82.5–88.9)
Specificity	58.9 (54.4–63.4)	65.3 (60.9–69.6)	54.9 (50.3–59.4)	50.7 (46.2–55.3)	55.6 (51–60.1)	46.5 (41.9–51.1)
Accuracy	83.1 (79.7–86.6)	85.7 (82.5–88.9)	81.5 (77.9–85.1)	76.3 (72.4–80.2)	77.1 (73.3–81)	75.8 (71.9–79.7)
Youden’s index	58.5 (54.0–63.1)	65.3 (60.9–69.6)	54.3 (49.7–58.8)	37.7 (33.3–42.2)	44.8 (40.3–49.4)	32.2 (27.9–36.5)
Precision	78.0 (74.2–81.8)	80.5 (76.8–84.1)	76.6 (72.7–80.5)	80.9 (77.3–84.5)	78.1 (74.3–81.9)	82.6 (79.1–86.1)
F1 Score	87.5 (84.5–90.6)	89.2 (86.3–92.0)	86.5 (83.4–89.7)	83.8 (80.5–87.2)	83.3 (79.9–86.8)	84.1 (80.8–87.5)
MCC	67.2 (62.9–71.5)	72.5 (68.4–76.6)	63.8 (59.4–68.2)	40.1 (35.6–44.6)	48.6 (44–53.2)	33.5 (29.2–37.9)
AUC	68.9 (64.7–73.2)	74.9 (70.9–78.9)	67.5 (63.2–71.8)	71.4 (67.2–75.5)	76.3 (72.4–80.2)	65.1 (60.8–69.5)

AUC: Area Under the Curve; TP: true positive; FN: false negative; FP: false positive; TN: true negative; MCC: Matthews correlation coefficient.

**Table 4:** OPG performance analysis according to the EFP/AAP 2018 and CDC/AAP 2012 case definitions (*n* = 456). Values are expressed as mean and 95% Confidence Interval (95% CI).

	EFP/AAP 2018			CDC/AAP 2012		
	Overall (*n* = 456)	High-Quality Radiography (*n* = 175)	Low-Quality Radiography (*n* = 281)	Overall (*n* = 456)	High-Quality Radiography (*n* = 175)	Low-Quality Radiography (*n* = 281)
AUC	76.6 (72.7–80.5)	80.7 (77.1–84.3)	74.2 (70.2–78.2)	75.9 (71.9–79.8)	77.2 (73.3–81)	75.2 (71.2–79.2)
**Staging Precision (95% CI)**						
No disease	51.9 (50.7–53.1)	65.3 (64.2–66.4)	43.4 (42.2–44.5)	61.8 (60.7–63)	74.5 (73.4–75.5)	52.4 (51.2–53.6)
Mild–Moderate	58.8 (57.6–59.9)	66.2 (65.1–67.3)	53.3 (52.1–54.4)	55.5 (54.3–56.7)	57.9 (56.7–59.1)	54.2 (53.1–55.4)
Severe	64.0 (62.8–65.1)	65.7 (64.6–66.8)	63.2 (62–64.3)	53.9 (52.7–55.1)	46.2 (45–47.3)	59.2 (58.1–60.4)

## Appendix A

**Table A1:** The Standards for Reporting Diagnostic Accuracy (STARD) recommendations.

Section & Topic	No	Item	Reported on Page
**Title or Abstract**			
	**1**	Identification as a study of diagnostic accuracy using at least one measure of accuracy (such as sensitivity, specificity, predictive values, or AUC)	1
**Abstract**			
	**2**	Structured summary of study design, methods, results, and conclusions (for specific guidance, see STARD for Abstracts)	1
**Introduction**			
	**3**	Scientific and clinical background, including the intended use and clinical role of the index test	1–2
	**4**	Study objectives and hypotheses	2
**Methods**			
Study design	**5**	Whether data collection was planned before the index test and reference standard were performed (prospective study) or after (retrospective study)	2
Participants	**6**	Eligibility criteria	2
	**7**	On what basis potentially eligible participants were identified (such as symptoms, results from previous tests, inclusion in registry)	2
	**8**	Where and when potentially eligible participants were identified (setting, location and dates)	2
	**9**	Whether participants formed a consecutive, random or convenience series	2
Test methods	**10a**	Index test, in sufficient detail to allow replication	2–3
	**10b**	Reference standard, in sufficient detail to allow replication	2–3
	**11**	Rationale for choosing the reference standard (if alternatives exist)	2–3
	**12a**	Definition of and rationale for test positivity cut-offs or result categories of the index test, distinguishing pre-specified from exploratory	NA
	**12b**	Definition of and rationale for test positivity cut-offs or result categories of the reference standard, distinguishing pre-specified from exploratory	NA
	**13a**	Whether clinical information and reference standard results were available to the performers/readers of the index test	NA
	**13b**	Whether clinical information and index test results were available to the assessors of the reference standard	NA
Analysis	**14**	Methods for estimating or comparing measures of diagnostic accuracy	3–4
	**15**	How indeterminate index test or reference standard results were handled	3–4
	**16**	How missing data on the index test and reference standard were handled	3–4
	**17**	Any analyses of variability in diagnostic accuracy, distinguishing pre-specified from exploratory	3–4
	**18**	Intended sample size and how it was determined	3–4
**Results**			
Participants	**19**	Flow of participants, using a diagram	4
	**20**	Baseline demographic and clinical characteristics of participants	for more details see [12,18]
	**21a**	Distribution of severity of disease in those with the target condition	4
	**21b**	Distribution of alternative diagnoses in those without the target condition	4–5
	**22**	Time interval and any clinical interventions between index test and reference standard	NA
Test results	**23**	Cross tabulation of the index test results (or their distribution) by the results of the reference standard	5–6
	**24**	Estimates of diagnostic accuracy and their precision (such as 95% confidence intervals)	5–6
	**25**	Any adverse events from performing the index test or the reference standard	NA
**Discussion**			
	**26**	Study limitations, including sources of potential bias, statistical uncertainty, and generalisability	6–8
	**27**	Implications for practice, including the intended use and clinical role of the index test	6–8
**Other Information**			
	**28**	Registration number and name of registry	NA
	**29**	Where the full study protocol can be accessed	NA
	**30**	Sources of funding and other support; role of funders	7
